# Identifying Elevated Risk for Future Pain Crises in Sickle-Cell Disease Using Photoplethysmogram Patterns Measured During Sleep: A Machine Learning Approach

**DOI:** 10.3389/fdgth.2021.714741

**Published:** 2021-07-26

**Authors:** Yunhua Ji, Patjanaporn Chalacheva, Carol L. Rosen, Michael R. DeBaun, Thomas D. Coates, Michael C. K. Khoo

**Affiliations:** ^1^Department of Biomedical Engineering, University of Southern California, Los Angeles, CA, United States; ^2^Department of Biomedical Engineering, Carnegie Mellon University, Pittsburgh, PA, United States; ^3^Department of Pediatrics, Case Western Reserve School of Medicine, Cleveland, OH, United States; ^4^Division of Hematology-Oncology, Department of Pediatrics, Vanderbilt University School of Medicine, Nashville, TN, United States; ^5^Department of Pediatrics, Children's Hospital Los Angeles, Cancer and Blood Disease Institute, University of Southern California Keck School of Medicine, Los Angeles, CA, United States

**Keywords:** sickle cell anemia, photoplethysmography, peripheral vasoconstriction, sleep, machine learning, vaso-occlusive crises

## Abstract

Transient increases in peripheral vasoconstriction frequently occur in obstructive sleep apnea and periodic leg movement disorder, both of which are common in sickle cell disease (SCD). These events reduce microvascular blood flow and increase the likelihood of triggering painful vaso-occlusive crises (VOC) that are the hallmark of SCD. We recently reported a significant association between the magnitude of vasoconstriction, inferred from the finger photoplethysmogram (PPG) during sleep, and the frequency of future VOC in 212 children with SCD. In this study, we present an improved predictive model of VOC frequency by employing a two-level stacking machine learning (ML) model that incorporates detailed features extracted from the PPG signals in the same database. The first level contains seven different base ML algorithms predicting each subject's pain category based on the input PPG characteristics and other clinical information, while the second level is a meta model which uses the inputs to the first-level model along with the outputs of the base models to produce the final prediction. Model performance in predicting future VOC was significantly higher than in predicting VOC prior to each sleep study (F1-score of 0.43 vs. 0.35, *p*-value <0.0001), consistent with our hypothesis of a causal relationship between vasoconstriction and future pain incidence, rather than past pain leading to greater propensity for vasoconstriction. The model also performed much better than our previous conventional statistical model (F1 = 0.33), as well as all other algorithms that used only the base-models for predicting VOC without the second tier meta model. The modest F1 score of the present predictive model was due in part to the relatively small database with substantial imbalance (176:36) between low-pain and high-pain subjects, as well as other factors not captured by the sleep data alone. This report represents the first attempt ever to use non-invasive finger PPG measurements during sleep and a ML-based approach to predict increased propensity for VOC crises in SCD. The promising results suggest the future possibility of embedding an improved version of this model in a low-cost wearable system to assist clinicians in managing long-term therapy for SCD patients.

## Introduction

Sickle cell disease (SCD) is an inherited blood disorder that results from an amino acid substitution in the beta globin chain of hemoglobin, producing sickle hemoglobin (1). When sickle hemoglobin polymerizes in the deoxygenated state, flexible discoid red blood cells are transformed into rigid sickle-shaped erythrocytes that can obstruct capillary blood flow if they fail to escape the microvasculature before the transformation occurs (2, 3). The clinical manifestation of these obstructions of microvascular flow is vaso-occlusive crisis (VOC), characterized by episodes of pain and subsequently organ damage or even more severe consequences (4, 5). In children and adolescents with SCD, the pain events are generally acute and occur intermittently with frequency increasing with age; whereas in adulthood, the acute events occur on top of chronic pain (6, 7). Since SCD individuals have low level, asymptomatic sickling all the time, it remains unclear how transient regional vaso-occlusion cascades into full-blown VOC. Much of the contemporary research in SCD has been focused on elucidating the underlying molecular and cellular factors that decrease microvascular flow by blocking the post-capillary venules (8). However, these processes are ongoing during steady state. Why or how transient regional vaso-occlusion cascades into large-scale VOC in response to certain stimuli remains unknown. There is also a significant amount of variability in VOC frequency and severity among SCD patients that is not fully explained by the biology of this progressive chronic vascular disease. However, there is growing evidence that the autonomic nervous system (ANS) plays an important role in triggering the onset of VOC (9–11). Of particular significance to subjects with SCD is the possibility that ANS-mediated vasoconstriction in the arterioles can reduce microvascular flow from already low basal levels, prolonging capillary blood transit time further and thus promoting regional vaso-occlusion.

Sleep-disordered breathing, and in particular obstructive sleep apnea, has been found to be prevalent among subjects with SCD (12, 13). For this reason, it has been thought that the intermittent hypoxia that results from nocturnal apnea or hypopnea would lead to higher frequency and severity of VOC in SCD subjects. But Willen et al. (14) found no relationship between low mean nocturnal SpO_2_ and incidence of VOC in a prospective multi-center cohort study of over 200 SCD children. Transient surges in sympathetic activity generally accompany the arousals and limb movements that occur during sleep in obstructive sleep apnea and periodic leg movement disorder, both of which are common in SCD (15, 16). These sympathetic surges lead to peripheral vasoconstriction and transient increases in heart rate that raise blood pressure (17). In SCD subjects, such events likely translate to recurring episodes in which microvascular blood flow is reduced, thus increasing the likelihood of triggering VOC. In a recently published analysis (18) of the same database of subjects in Willen et al's. (14) study, we identified a significant association between the median magnitude of vasoconstriction (M_vasoc_), inferred from the finger photoplethysmogram (PPG) during sleep, and the rate of subsequent VOC. An interesting secondary finding was that the indices reflecting frequency of arousal, limb movements and obstructive apnea/hypopnea were all significant predictors of M_vasoc_ but none of these demonstrated a significant association with VOC pain rate. This result suggests that, although the sympathetic surges that accompany disordered sleep contribute to episodes of peripheral vasoconstriction, it is the collective effect of these events, quantified by M_vasoc_, that best represents their net impact on microvascular blood flow and the consequent likelihood for triggering large-scale VOC.

While our previous study (18) was aimed at identifying the key physiological factors linking vasoconstriction to VOC pain frequency, the present work focuses on developing algorithms to improve the ability to predict future likelihood of VOC. This is achieved by employing an approach that uses a two-level stacking machine learning (ML) model to recognize features extracted from the complex dynamic patterns of the PPG and heart rate signals that occur in conjunction with episodic nocturnal vasoconstrictions.

The rest of this paper is structured as follows. The Methods section describes: (1) the input features that we extracted from overnight polysomnogram signals and how we defined VOC pain rates, (2) the process of feature selection to be included in the model, (3) the development of models from single-level bases to two-level stacking model, (4) the cross-validation process and (5) the chosen evaluation metrics. The Results section compares the performances of the base models and the two-level stacking model with a “baseline” random model. Finally, the Discussion section summarizes the key findings from the application of a machine learning approach to data derived from non-invasive measurements of heart rate and finger photoplethysmogram during sleep, with the goal of predicting future VOC crises in SCD subjects. The study limitations and implications for home-based clinical application are also discussed.

## Methods

### Data Analysis

As in our previous study (18), our analyses were conducted using the same database derived from the multi-center Sleep and Asthma Cohort (SAC) study of children and adolescents with SCD (14). Data from 252 SCD subjects were considered for analysis: 240 subjects were homozygous for sickle cell hemoglobin [HbSS], and 12 were compound heterozygous for sickle β zero thalassemia [HbSβ0]. Overnight polysomnograms (PSG) were obtained on all participants in the SAC. Deidentified digital recordings of all channels from each PSG and clinical datasets from 212 participants were selected for our analyses based on the quality of the polysomnogram recording and completeness of the records on pain events. Further details about the recruitment and consent process in the SAC are given in Willen et al. (14).

The information used for detection and quantification of peripheral vasoconstriction episodes during sleep was derived from two channels of the PSG recording: (1) the sequences of R-peaks extracted from the electrocardiogram, from which we determined the R-to-R interval (RRI) on a beat-to-beat basis over the entire sleep duration; (2) the PPG waveform acquired from pulse oximetry (Masimo SET (v2), Irvine, CA). The R-peaks were also used as time-markers for delineating the corresponding cardiac pulses in the PPG waveform. From each PPG pulse, we derived the peak to trough amplitude (PPGa). Both PPGa and RRI signals took the form of uniformly sampled time series using zero-order hold interpolation, which were downsampled to 2 Hz by the method introduced by Berger et al. (19). Segments with artifacts due to motion, interruptions to the sleep study, signal clipping, or abrupt changes in gain of the recording channel were excluded from further analysis. Ectopic beats (determined by RRIs that are longer or shorter than 3.5 standard deviations from mean) were interpolated. PPGa signals were normalized with respect to the 95-percentile maximum value (from sleep onset to end of sleep) for each subject's sleep study. Respiratory influences in both signals were removed using a low pass filter with cutoff frequency of 0.18 Hz. Vasoconstriction events were detected using the signal processing algorithm detailed in Chalacheva et al. (18). After detecting the vasoconstriction events, the resampled (2 Hz) PPGa and RRI signals around the events were selected such that each segment was 180 s in total duration. This parameter (segment duration) was based on preliminary analyses of the durations of vasoconstrictions in randomly selected subjects, in which we found that the vast majority of vasoconstriction events lasted much <150 s following onset. Thus, the first 30 s of each segment prior to vasoconstriction onset were used to represent the pre-vasoconstriction baseline for that segment. The features associated with vasoconstriction events were derived from the dynamics displayed in all these segments (see next section, “Feature Selection”). In addition, all extracted segments, each of fixed duration of 180 s, were used as inputs to the convolutional neural network (CNN) base model (see “Model Development” section). Given the resampling rate of 2 Hz, the CNN model thus received a total of 720 inputs (180 × 2 samples × 2 channels). Further details of the CNN model architecture are provided in Supplementary Material.

Hospitalization events of the subjects resulting from severe pain induced by VOC were recorded in the SAC study. These records were used to derive the pain rate, quantified by the average number of pain episodes (that require hospitalization) per year. “Post-PSG pain rate” was defined as average pain rate following the PSG study till the end of the SAC study for each individual. “Pre-PSG pain rate” was defined as average pain rate before the PSG study. A threshold of 1.5 pain episodes per year was chosen to separate the subjects into high pain (≥1.5) and low pain (<1.5) categories. Applying this criterion to post-PSG pain rate, 36 subjects were categorized as “high pain,” and 176 subjects were categorized as “low pain.” The same criterion, applied to pre-PSG pain rate, yielded 31 “high pain” and 181 “low pain” individuals. A categorical dependent variable (pain category) was chosen over a continuous one (pain rate) for three reasons. First, in the SAC study, 5% of the subjects contributed toward ~25% of all pain episodes. Secondly, from a clinical standpoint, it is more important to distinguish the SCD patients with 2 pain episodes per year from the patients with no episode, than to distinguish the patients with 6 episodes per year from the ones with 2 per year. Employing a categorical framework for characterizing VOC frequency serves this goal better than treating pain rate as a continuous variable. Thirdly, only 212 subjects were studied in this work – this is a relatively small dataset in the context of machine learning. Adopting pain category over pain rate can limit the impact of outliers (extremely high pain rate) on the machine learning models, reduce the amount of overfitting, and allow the models to be trained more effectively.

All vasoconstriction events belonging to the same subject shared the same label: the pain category of that subject.

### Feature Selection

**Figure 1:**
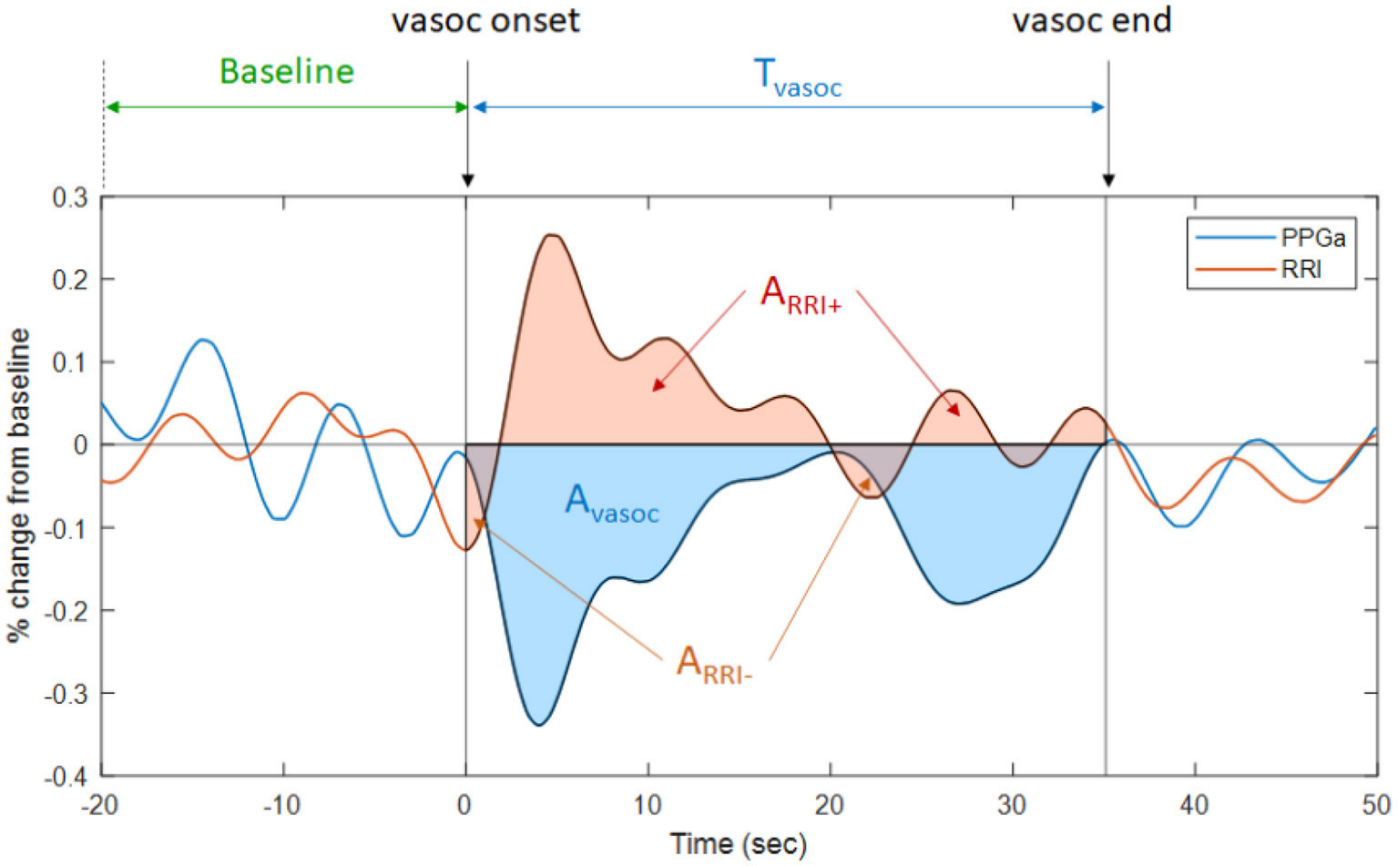
Illustration of the changes in PPGa (blue tracing) and RRI (brown tracing) that occur during a vasoconstriction episode. T_vasoc_ represents the duration between the onset and end of a vasoconstriction episode. A_vasoc_ (blue-shaded area) represents the cumulative amount of vasoconstriction over T_vasoc_. The average magnitude of vasoconstriction for this episode, M_vasoc_, is calculated as the ratio between A_vasoc_ and T_vasoc_. Thus, M_vasoc_ is also the average percent change in PPGa from pre-vasoconstriction baseline. A_RRI−_ is the RRI area below baseline during vasoconstriction (i.e., cumulative amount of heart rate increase during this vasoconstriction episode). A_RRI+_ is the RRI area above baseline (i.e., cumulative amount of heart rate decrease during vasoconstriction episode). M_vasoc_, A_RRI−_, A_RRI+_, and total number of vasoconstriction episodes (N_vasoc_, not shown in figure) were selected as features representing vasoconstriction dynamics.

The inputs that were used by the ML model to predict VOC pain category were selected from a pool of 44 candidate features derived from five groups of information: (a) clinical data and subject characteristics, (b) sleep-related indices, (c) all-night RRI and heart rate variability indices, (d) all-night PPGa statistics, and (e) compact descriptors of the dynamic fluctuations in PPGa and RRI associated with detected vasoconstrictions. The final and reduced set of features employed in the subsequent analyses was determined by a screening procedure that sought to: (a) maximize the association between each feature and pain category, and (b) minimize the pairwise Spearman's correlation coefficient between that feature and other candidate features in the same feature group. The selected set of 20 features is listed in Table 1. The entire set of 44 candidate features that we initially screened and the procedures we employed to select the final 20 features for use as inputs are given in Supplementary Table 1. Figure 1 provides a graphic illustration of the key features associated with RRI and PPGa changes (Table 1) during a vasoconstriction event (see figure legend for details).

**Table 1:** Feature selection.

**Feature/input variable**	**Feature type**
Age	Clinical/Subject
Sex	Clinical/Subject
Hemoglobin	Clinical/Subject
White blood cell count	Clinical/Subject
Reticulocyte count	Clinical/Subject
Neutrophil count	Clinical/Subject
Diastolic blood pressure	Clinical/Subject
Systolic blood pressure	Clinical/Subject
Body mass index	Clinical/Subject
Hydroxyurea use	Clinical/Subject
Arousal index^a^	Sleep
Apnea-hypopnea index^b^	Sleep
Number of limb movements	Sleep
Effective sleep duration^c^	Sleep
Median of RRI mean per 5 min	All-night RRI/HRV
Median of PPGa CV per 5 min	All-night PPGa
Median of M_vasoc_ values from all detected vasoconstriction events in sleep study	Vasoconstriction
Median of A_RRI−_^d^ values from all detected vasoconstriction events in sleep study	Vasoconstriction
Median of A_RRI+_^e^ values from all detected vasoconstriction events in sleep study	Vasoconstriction
N_vasoc_: total number of vasoconstriction events in sleep study	Vasoconstriction

a *Average number of arousals per hour of sleep*.

b *Average number of apneas and hypopneas per hour of sleep*.

c *Total duration of sleep during PSG, minus (short) durations in which PPG signal was corrupted by artifact or noise*.

d *RRI area below baseline during vasoconstriction (see Figure 1)*.

e *RRI area above baseline during vasoconstriction (see Figure 1)*.

### Model Development

In the first phase of model development, we evaluated the ability of a variety of stand-alone ML algorithms (“base models”) to predict pain category of the subjects using the set of 20 selected features, as described earlier. These algorithms included: (1) multiple logistic regression, (2) support vector machine (SVM) with linear kernel (20), (3) SVM with radial basis function (RBF) kernel, (4) random forest (21), (5) extremely randomized trees or ExtraTrees (22), (6) adaptive boosting or AdaBoost (23), (7) one-dimensional CNN (24), (8) multi-layer perceptron (25), (9) SVM with polynomial kernel (20), (10) gradient boosting trees (26, 27), and (11) extreme gradient boosting trees or XGBoost (28). The CNN base model accepted all segments (each of 180 s duration) extracted from the PPGa and RRI time-series as inputs, while all other 10 base models used the 20 selected features. The output of each of these base models was pain category.

Table 2 compares the performance of all the base models that we tested, using the F1 metric (see Evaluation Metric section later). Three of these base models (SVM with polynomial kernel, gradient boosting trees, and XGBoost) were rejected from being included in the subsequent two-level stacking model since they performed significantly worse than all the other base models (Table 2) and also no better than the negative binomial model used in our previous analysis (Table 3).

**Table 2:** Performance of base models.

**Model**	**F1-score**	**95% CI**
Logistic regression	0.3751	[0.3594, 0.3907]
SVM with linear kernel	0.3855	[0.3713, 0.3997]
SVM with RBF kernel	0.3726	[0.3605, 0.3846]
SVM with polynomial kernel	0.2391	[0.2149, 0.2633]
Random forest	0.3617	[0.3420, 0.3813]
ExtraTrees	0.3775	[0.3645, 0.3904]
AdaBoost	0.3534	[0.3370, 0.3699]
Gradient boosting trees	0.2151	[0.1891, 0.2411]
XGBoost	0.3101	[0.2937, 0.3265]
CNN	0.3608	[0.3503, 0.3713]
MLP	0.4147	[0.4033, 0.4261]

*Inputs are 20 selected features. SVM, support vector machine; CNN, convolutional neural network; MLP, multi-layer perceptron*.

**Table 3:** Model performance comparison.

**Model**	**Inputs to meta-model**	**Output (pain category)**	**F1-score**	**95% confidence intervals**
Random Case (Max F1)	N/A	N/A	0.2903	N/A
Negative Binomial	Age, Hgb, Mvasoc	Post-PSG	0.3279	N/A
(A) Full Model	20 features + 7 base models	Post-PSG	0.4255	[0.4183, 0.4327]
(B) No direct features to Meta-Model	7 base models	Post-PSG	0.3827	[0.3695, 0.3960]
(C) Features only + no base models to Meta-Model	20 features	Post-PSG	0.4147	[0.4033, 0.4261]
(D) M_vasoc_ excluded from features	19 features + 7 base models	Post-PSG	0.4155	[0.4041, 0.4269]
(E) Pre-PSG pain category	20 features + 7 base models	Pre-PSG	0.3505	[0.3377, 0.3633]

**Figure 2:**
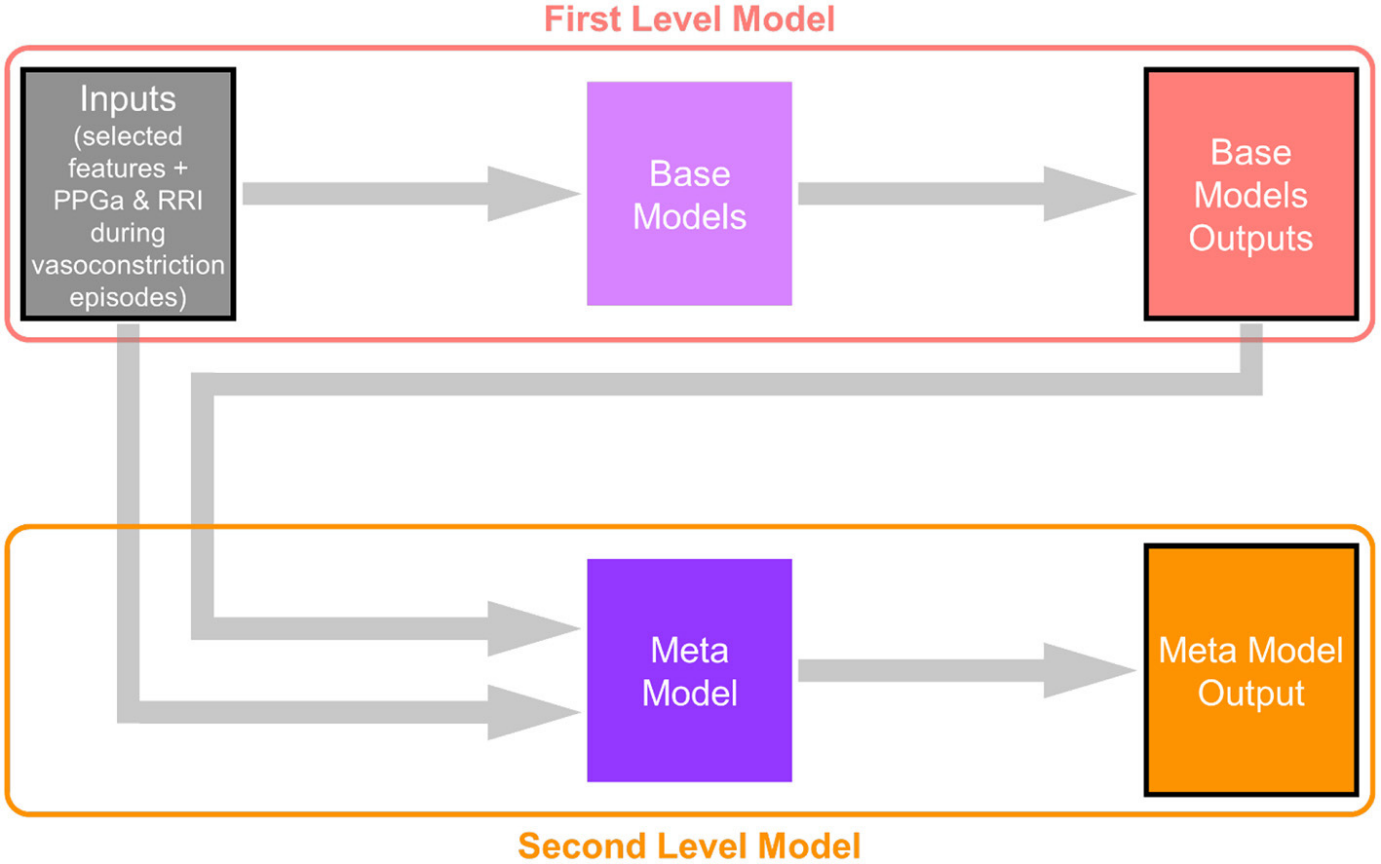
Architecture of the two-level stacking model. The base models in the first level take subjects' physiological and ANS features as input (the CNN base-model takes PPGa and RRI time-series around the detected vasoconstriction events). The meta model in the second level takes both subject features and the outputs of base models (prediction of subjects' pain categories) as inputs, and makes the final prediction.

The multi-layer perceptron (MLP) performed substantially better than all the other base models, and thus was selected to be the algorithm representing the “meta model” in the second level of the stacking model. The second level meta model took the outputs of all base models, plus the 20 selected features, as inputs. The output of the second level of the stacking model was the final prediction for subject pain category. Figure 2 shows the architecture of this two-level stacking model. The human analogy of the two-level stacking model would be that the first-level base models represent experts with specialized skills that report to a higher-level “supervisor” (represented by the second-level meta model). The “supervisor” considers all the decision calls submitted by the base models and also directly evaluates the information derived from the features to render the final judgement of what pain category to assign to each subject.

Further details about the CNN and MLP models can be found in Supplementary Material.

### Cross-Validation

**Figure 3:**
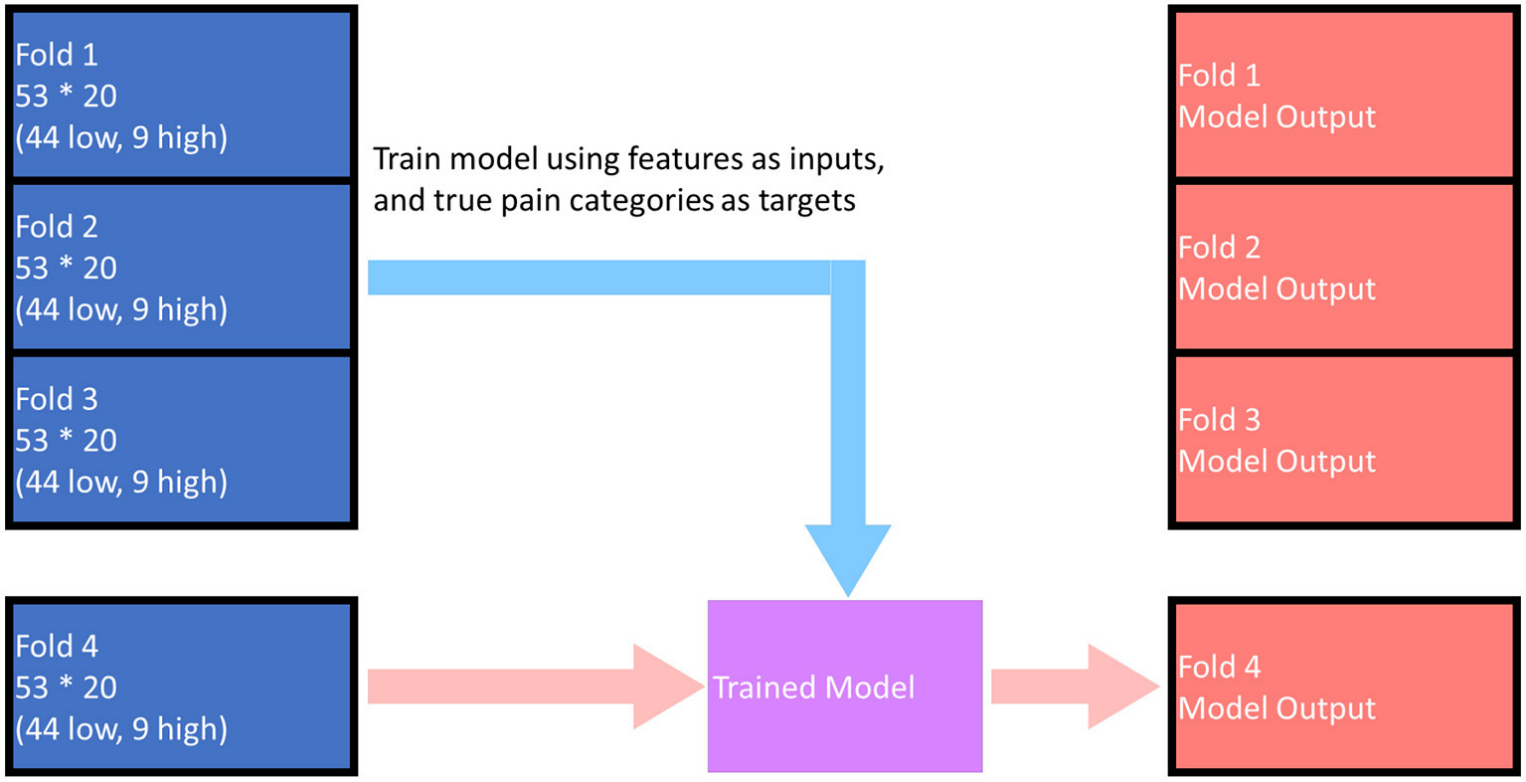
Illustration of the cross-validation training and evaluation process. The above figure demonstrates the process for *i* = 4, when model outputs for fold 1 ~ fold 3 has already been calculated. The model outputs for each fold (any one of the red folds in the figure) were predicted by the model trained by data in the other 3-folds. The overall performance of the model was evaluated based on the red folds in the figure.

The base models and the meta model were all trained and evaluated using *k*-fold cross-validation; in this case, the parameter “*k*” was chosen to be 4. The cross-validation process can be described as follows (as illustrated in Figure 3).

- The subjects were shuffled using random number generator (controlled by a seed) and divided into 4 sets (“folds”). Each fold contained 53 subjects (44 low pain, 9 high pain).
- The *i-th* fold (1 ≤ *i* ≤ 4) was selected as the test fold, and while the other 3-folds were used as training folds.
- The model was trained using data from the training folds, model performance was assessed using data from the test fold (fold “*i*”), and the results (both model outputs and evaluation) were saved to the output pool.
- Steps 2 and 3 were repeated with each of the other folds being used as the test fold.
- Following execution of all 4-folds, the output pool contains all the predicted model outputs and the performance evaluation numbers for all the subjects.

**Figure 4:**
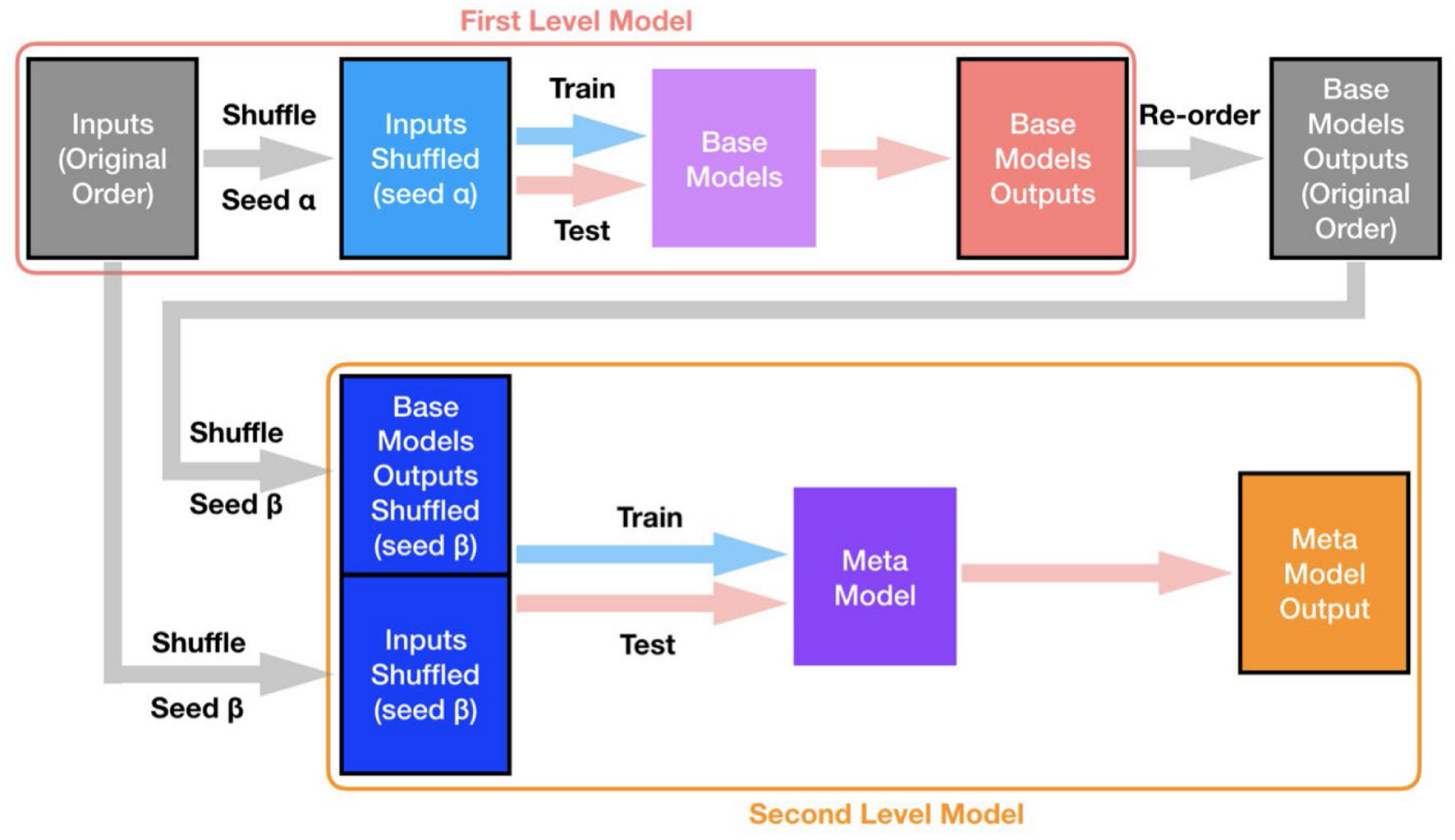
Overall process for training and evaluation of the two-level stacking model. Both base models and the meta model were all trained and evaluated in the cross-validation manner. The shuffling process (to split subjects into different folds for cross-validation) for base models is controlled by a certain random seed α (shared for all base models), while the shuffling process for the meta model is controlled by a different random seed β.

The base models were first trained in the cross-validation manner, and the shuffling process was initiated using a random seed α. Then the outputs of base models were re-ordered (based on subjects) to the original order of the dataset. Next, the meta model was also trained in the cross-validation manner, and the shuffle process for both the features (original order) and the outputs of base models (original order) was governed by another random seed β. A schematic illustration of the overall process for cross-validation and evaluation of the two-level stacking model is displayed in Figure 4.

### Evaluation Metric

Since there were two output categories (“high pain” and “low pain”), we classified those subjects in the high pain category to be “true positive” (TP) if they were actually high pain subjects and predicted to have high pain. Those subjects who actually had high pain but predicted to be low pain fell into the “false negative” (FN) category. Subjects who were actually low pain and predicted to have low pain constituted the “true negative” (TN) category. Finally, subjects who were low pain but predicted to have high pain were classified as “false positive” (FP). The two key metrics for evaluating the performance of prediction algorithms are “true positive rate,” also known as “recall” or “sensitivity,” defined as TP/(TP+FN), and “positive predictive value,” also known as “precision,” defined as TP/(TP+FP). A common alternate measure is “false positive rate,” defined as FP/(FP+TN), also referred to in the statistical literature as “type 1 error.” The complement of false positive rate is “specificity” [= TN/(FP+TN)]. There is usually a trade-off between recall and precision or false positive rate, particularly when the data are imbalanced, as in our case since there are many more subjects with “low pain” than those with “high pain.” Thus, we employed a measure that combines these key metrics (29). We chose the broadly accepted F1-score, which is the harmonic mean of recall and precision:


$$\begin{array}{l} F1\;=\;\frac{2\;precision^{*}recall}{\left(precision+recall\right)} \end{array}$$

In order to reduce the impact of randomness introduced in the subject shuffling procedures, the two-level stacking model underwent training 20 times, with different random seeds controlling the shuffling procedures in different trials. The mean F1-score of the 20 trials was taken as the performance of the model. F1-score was chosen over curve-based metrics (such as ROC curve or AUC) since curve-based metrics require the model to output the probability for a subject to be positive (high pain), which is difficult to define when the model is trained multiple times. Additionally, it has been shown that measures such as accuracy, ROC curve or AUC may present an overly optimistic evaluation of the model with imbalanced data (30, 31). F1-score takes into account both precision and recall and thus was chosen as the more appropriate evaluation metric of our model over the ROC curve.

## Results

### Sample Datasets

**Figure 5:**
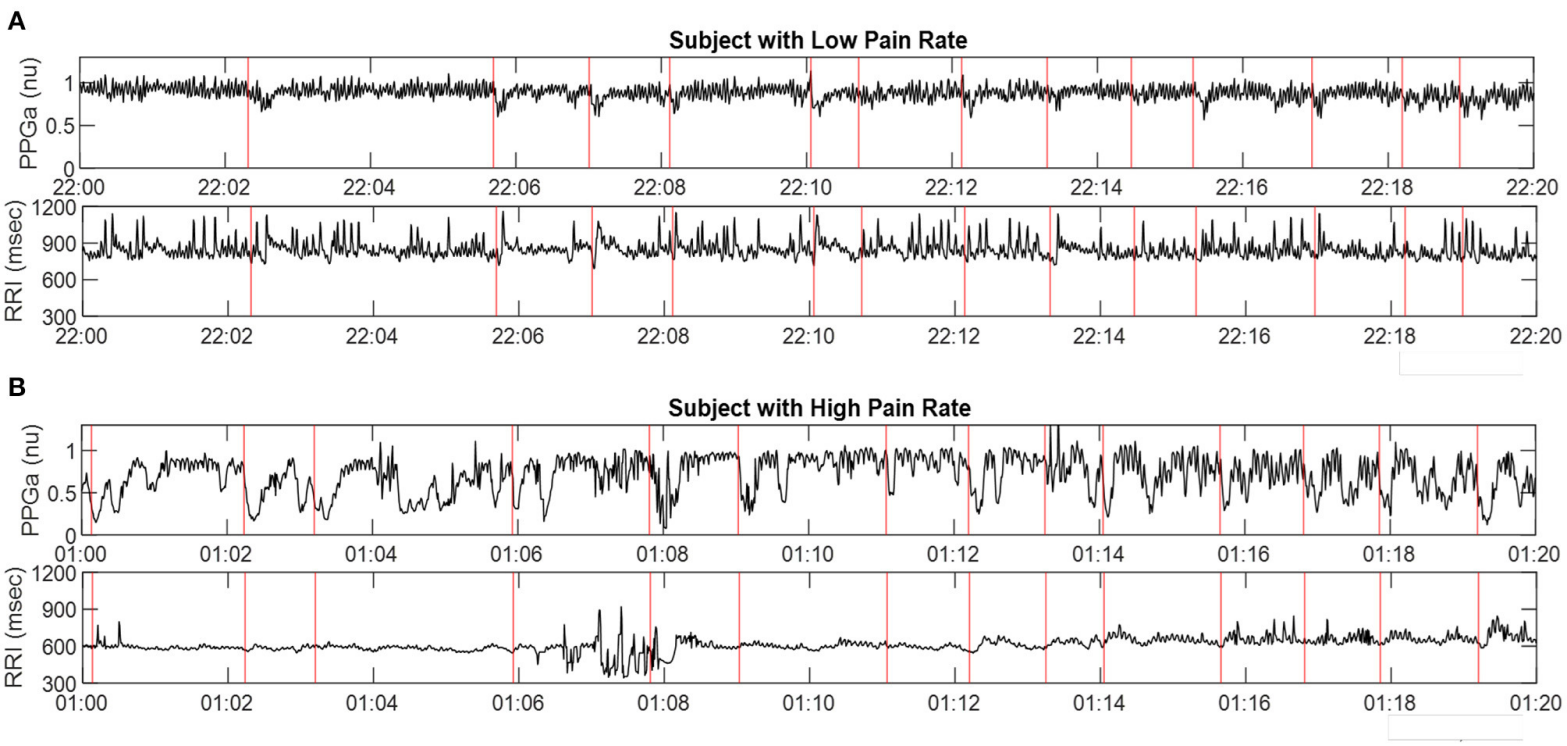
Representative samples of PPGa and RRI time-series (20-min sections) derived from PSG data in two subjects: **(A)** in low pain category, and **(B)** in high pain category. Red vertical lines represent detected start-times of significant vasoconstrictions. The time-axes in A and B are displayed in military time format.

Examples of 20-min segments selected from the overnight beat-to-beat PPGa and RRI time-series of 2 subjects (A and B) are displayed in Figure 5. The red vertical lines in all 4 plots represent the times at which significant vasoconstrictions, as detected by the algorithm of Chalacheva et al. (18), were considered to have begun. In both cases displayed, each red line is accompanied by a noticeable drop in PPGa. There are a number of significant vasoconstrictions that may have been missed by the automated detection algorithm, due in many cases to repetitive vasoconstrictions that were close together and the lack of a sufficiently long, relatively flat baseline prior to the missed vasoconstriction. Subject A had a post-PSG pain rate of 0 events/year, while Subject B's pain rate was 7.2 events/year. The median M_vasoc_ for Subject A was 13% while median M_vasoc_ for Subject B was 27%. Both subjects had similar values for arousal index, the number of arousals per hour of sleep, (A: 8.4 h^−1^, B: 9.5 h^−1^) and low obstructive apnea-hypopnea index or AHI, the number of apnea and hypopnea events per hour of sleep (A: 1.1 h^−1^, B: 0.12 h^−1^).

### Benchmarks for Least Acceptable Model Performance

We performed calculations with a random model to provide the benchmarks that represent the minimum acceptable levels of model performance. Since the problem involved only 2 output labels (“low pain” vs. “high pain”), we simulated the tossing of a coin with different head-tail probabilities. Suppose the probability of obtaining a head (representing “high pain”) is *p*. The SAC dataset contains 212 subjects, with 36 belonging to the positive category (“high pain”), and 176 belonging to the negative category (“low pain”) based on post-PSG pain rate. Thus, the expected confusion matrix for this random model would contain the following entries:

TP = 36*p*, FN = 36 (1 – *p*), FP = 176*p*, TN = 176 (1 – *p*)

The corresponding precision, recall and F1-score values could be calculated as defined in the formulas listed in the “Evaluation Metrics” section.

We considered 3 cases:

- Here, we simulated a fair coin that yielded predictions of “high pain” and “low pain” with equal probability (*p* = 0.5). This resulted in F1 = 0.2535.
- Given the unbalanced distribution of “high pain” (36) vs. “low pain” (176) subjects, here we examined the consequences of a random model with *p* = 36/212, i.e., the probability of obtaining “high pain” with a single toss is <0.17. This gave an F1-score of 0.1667.
- In this third “random” scenario, we simulated a coin toss that was totally biased toward “high pain,” with *p* = 1. This led to F1 = 0.2903. It can be shown that this is the highest possible F1-score that can be derived from the random model. It also represented the lowest acceptable F1-score that any of the ML models we tested had to exceed in order to have a level of performance that was “better than random.”

### Performance of the Two-Level Stacking Model

Table 3 compares the performance of the random model (best possible performance), the negative binomial model (18), and different variations of the two-level stacking model (labeled “A” through “E”), using the F1-score metric.

- The “full model,” consisting of the two-level stacking model with 7 base models utilizing all 20 features, and including the time-series of PPGa and RRI as inputs to the CNN base model, yielded the best outcome, with average F1-score of 0.4255. This predictive capability was substantially higher (F1 = 0.4255 vs. 0.3279) than what we had previously reported in Chalacheva et al. (18), using median M_vasoc_, age and hemoglobin level as predictors.
- In the reduced version of the two-level stacking model where the second-level meta model received only the output “decisions” of the 7 base models but not the 20 features directly, performance was substantially lower than that of the “full model,” but this was still better than the performance of the negative binomial model (0.3827 vs. 0.3279).
- In the other reduced version of the model where the inputs to the meta model consisted only of the 20 features but not the outputs of the 7 base models, the average F1 score was marginally lower than that attained by the “full model” (0.4147 vs. 0.4255), but significantly higher than Case B above (0.4147 vs. 0.3827).
- Here, we tested the scenario in which the full model was used, but a key vasoconstriction feature (M_vasoc_) was excluded from the information sent to the base and meta models. This reduced model displayed marginally lower performance relative to the “full model” (0.4155 vs. 0.4255) but the performance was similar to that in Case (C).
- The full two-level stacking model was also used to predict pre-PSG pain category. As demonstrated in Table 3, the F1-score for predicting pre-PSG pain was substantially worse than that for predicting post-PSG pain (0.3505 vs. 0.4255). This result is consistent with the finding reported by Chalacheva et al. (18) that the direction of causality is for high vasoconstriction propensity to predispose to future high VOC pain rate, rather than for high prior pain rate to influence current propensity for vasoconstriction.

## Discussion

### Summary and Interpretation of Key Findings

The application of machine learning techniques of different levels of complexity to the analysis of physiological signals has become ubiquitous in recent years. The application areas span the broad spectrum of biomedicine, ranging from medical imaging (32) and electrocardiogram analysis (33) to automated staging of sleep (34, 35), as well as the prediction or detection of sleep apnea (36). The present study represents the first attempt ever to apply a machine learning (ML) approach to data derived from non-invasive measurements of RRI (heart rate) and finger PPG during sleep, with the goal of predicting future VOC crises in SCD subjects. We developed a ML model with relatively simple architecture for this purpose, given the paucity of measurements of VOC pain in the small database (212 subjects) that was available for analysis. Nonetheless, we obtained valuable insights from this pilot study. The key findings derived from our analyses may be summarized as follows:

- Preliminary screening of all possible “inputs” to be used in our ML algorithms led to the identification of 20 features, which included clinical information, sleep-related indices, overall statistics of the heart-rate and PPGa time-series, and descriptors related to the vasoconstriction events. These features included M_vasoc_, age and hemoglobin level, the three parameters that were found in our previous study (18) to be significant predictors of post-PSG pain rate.
- We tested a total of 11 ML algorithms as stand-alone prediction models of pain category using the selected 20 features as inputs, with the exception of the CNN algorithm which used as inputs the segments of RRI and PPGa (of 180 s duration each) straddling all detected the vasoconstriction events. Based on relative performance, seven of these algorithms were selected as base models filling the first layer of a two-level stacking model. The best performing stand-alone algorithm (MLP) was selected to be the second-level “meta model,” the output of which was the overall model's prediction of pain category.
- The two-level stacking ML model (“full model”) that we developed performed much better than the negative binomial regression model used in Chalacheva et al. (18), as well as all other algorithms that used only the base-model outputs for predicting pain outcomes without the second tier meta model. We believe the modest F1 score of the “full model” was due in part to the relatively small database with substantial imbalance (176:36) between low-pain and high-pain subjects, as well as other factors influencing the development of VOC not captured by the PSG or clinical data.
- Performance of the “full model” in predicting post-PSG VOC pain category was significantly higher in comparison to predicting pre-PSG pain category, consistent with our hypothesis of a causal relationship between vasoconstriction and future pain incidence, rather than past pain leading to greater propensity for vasoconstriction.
- Eliminating M_vasoc_ as one of the inputs degraded the F1 score of the “full model,” but prediction performance remained higher than that attained by the negative binomial model (18). Our interpretation of this finding is that the other three vasoconstriction features and the median of the coefficients of variation of 5-min segments of PPGa provide complementary information about vasoconstriction dynamics that partially compensate for the absence of M_vasoc_ as one of the inputs.
- Although the two-level stacking ML model was superior in predictive capability relative to all other models that were tested in this study, the more conventional statistical approach that was adopted using the negative binomial model (18) provided a complementary perspective in allowing us to determine which features were the most significant predictors of post-PSG pain rate. Table 4 provides a more detailed comparison of these two models, based on the other performance metrics (precision, recall, specificity, accuracy) along with F1-score. The substantially higher sensitivity (recall) of the full model vis-à-vis the negative binomial model is noteworthy, showing that the former is superior in its ability to detect subjects with high pain, even though this results in a higher false positive rate (lower specificity). The imbalanced nature of the data (much larger fraction of subjects with low pain), on the other hand, allows the negative binomial model to more easily identify the true negatives – hence, the higher values of specificity and accuracy.

**Table 4:** Detailed comparison of performance metrics: full model vs. negative binomial model.

**Model performance metric(mean values)**	**Full model**	**Negative binomial**
F1-score	0.4255	0.3279
Precision	0.2915	0.4000
Recall (sensitivity)	0.7917	0.2778
Specificity	0.6048	0.9148
Accuracy	0.6366	0.8066

### Limitations

There were a number of important limitations that need to be addressed in future work. First, the size of the dataset (212 subjects) was relatively small in the context of studies that utilize machine learning. In addition, the features used for training the model were derived from the RRI and PPGa signals of only one PSG study per subject. Predicting pain rate a few years into the future for each subject based on data obtained from only one night of measurements clearly presupposes that this single study was representative of the subject's sleep patterns, and sleep-disordered breathing status would remain relatively stable over time. However, there is some support for this assumption based on a report by Mullin et al. (37), who found little change in the polysomnograms of a subset of this same cohort of SCD subjects over durations lasting 1 to 2-1/2 years. Another consequence of the small number of datasets is that unbiased evaluation using data that was reserved only for testing, but not for training, was not feasible. Some extent of “data leakage” was inevitable in the cross-validation process. This likely biased the hyper-parameter tuning and evaluation toward overfitting. The effect was reduced by applying different random seeds in hyper-parameter tuning and evaluation, but the leakage effect could not be thoroughly removed.

Another limitation was the unbalanced ratio of number of subjects in the high-pain category vs. the number of low-pain subjects (1:4.9 for post-PSG pain category and 1:5.8 for pre-PSG pain category). This issue was handled to some extent by assigning larger class weights to the positive samples (associated with high pain) in training and using F1-score as evaluation metric. As well, during 4-fold cross-validation, we constrained each of the 4 “folds” (each containing 53 samples) to contain nine high-pain subjects and 44 low-pain subjects, so that the proportion of high-pain to low-pain subjects in the overall dataset was mirrored in each fold. In spite of these mitigating measures, model performance was likely reduced due to lack in diversity of the features in the samples associated with high pain.

As well, the feature selection process applied in this work was based on similarity of distribution between a single independent variable (feature) and the dependent variable (pain category), which did not properly consider potential interaction effects among features. The vasoconstriction detection algorithm introduced in Chalacheva et al. (18) was critical to this work, as it was used to derive the features related to vasoconstriction events. However, the algorithm contains assumed settings that, in principle, could be tuned as hyper-parameters to further improve prediction outcomes. Examples of these parameters include the thresholds used for detection of “significant” vasoconstriction and the durations of the time-windows used for determining the times of onset and end of each vasoconstriction episode. As exemplified in Figure 5, there were likely many vasoconstriction episodes that were not detected or falsely detected by this automated algorithm due to aberrant behavior of the PPG signal.

### Implications for Home-Based Clinical Application

The promising results arrived at in this study point to the feasibility of designing a wearable system that provides a marker for the propensity to have more frequent VOCs. Although our analyses were conducted using data collected during overnight polysomnography, the key signal that we used to identify and quantify vasoconstrictions was the finger photoplethysmogram. Beat-to-beat pulse interval (or heart rate) can be derived from the PPG signal. Many commercial devices already measure PPG for continuous tracking of health and fitness, although most extract PPG signals from the wrist rather than the fingertip. In the present work, VOC pain rate for the SCD patients was derived from hospitalization records and clearly excluded pain episodes that were experienced in the home that were not sufficiently severe or persistent to motivate the patient to seek hospital care. Since most VOC pain is managed at home, we recently monitored self-reports of the frequency and intensity of pain and mental stress in SCD patients over a 13-month period using a mobile application (38). We found that greater pain intensity was associated with higher stress level after adjusting for age and gender. Also, it is well-known that many SCD patients report experiencing an “aura” that precedes the onset of VOC. In our mobile application study, >80% of the reports of aura were followed by pain. In another study (39), the sleep patterns of pediatric SCD subjects were monitored over 2 weeks using a wrist-mounted actigraphy device, and these were found to be associated with the occurrence of next-day pain recorded by daily pain diary. Taken together, these exciting recent developments point to the future possibility of embedding an ML-based model in a low-cost wearable system, consisting of a wristband paired with mobile phone, to assist clinicians in managing long-term therapy for SCD patients.

## Data Availability Statement

The original contributions presented in the study are included in the article/Supplementary Material, further inquiries can be directed to the corresponding author.

## Ethics Statement

The studies involving human participants were reviewed and approved by Institutional review boards of Washington University School of Medicine, Case Western Reserve University, and University College, London (data collection was part of a multi-center cohort study). Written informed consent to participate in this study was provided by the participants' legal guardian/next of kin.

## Author Contributions

YJ and PC processed the raw physiological signals, extracted features, and conducted preliminary statistical analyses. YJ developed the machine learning models and applied them to the data. YJ, MK, and TC interpreted the results. YJ and MK wrote the manuscript. PC, TC, and CR critically reviewed and edited the manuscript. MK and TC conceived data analysis ideas and provided guidance throughout. MD was the principal investigator of the SAC project and designed the concepts for SAC. CR contributed to the development of the SAC project, study concepts, and procedures. All authors contributed to the article and approved the submitted version.

## Conflict of Interest

The authors declare that the research was conducted in the absence of any commercial or financial relationships that could be construed as a potential conflict of interest.

## Publisher's Note

All claims expressed in this article are solely those of the authors and do not necessarily represent those of their affiliated organizations, or those of the publisher, the editors and the reviewers. Any product that may be evaluated in this article, or claim that may be made by its manufacturer, is not guaranteed or endorsed by the publisher.

## References

[1] Pauling L Itano HA Singer SJ Wells IC. Sickle cell anemia, a molecular disease. Science. (1949) 110:543–8. 10.1126/science.110.2865.543 15395398

[2] Eaton WA Hofrichter J Ross PD. A possible determinant in sickle cell disease. Blood. (1976) 47:621–7. 10.1182/blood.V47.4.621.621 1260125

[3] Eaton WA Bunn HF. Treating sickle cell disease by targeting HbS polymerization. Blood. (2017) 129:2719–26. 10.1182/blood-2017-02-765891 28385699 PMC5437829

[4] Ballas SK. Pain management of sickle cell disease. Hematol Oncol Clin North Am. (2005) 19:785–802. 10.1016/j.hoc.2005.07.008 16214644

[5] Darbari DS Sheehan VA Ballas SK. The vaso-occlusive pain crisis in sickle cell disease: definition, pathophysiology, and management. Eur J Haematol. (2020) 105:237–46. 10.1111/ejh.13430 32301178

[6] Brandow AM Zappia KJ Stucky CL. Sickle cell disease: a natural model of acute and chronic pain. Pain. (2017) 158:S79–S84. 10.1097/j.pain.0000000000000824 28221286 PMC5350013

[7] Uwaezuoke SN Ayuk AC Ndu IK Eneh CI Mbanefo NR Ezenwosu OU. Vaso-occlusive crisis in sickle cell disease: current paradigm on pain management. J Pain Res. (2018) 11:3141–50. 10.2147/JPR.S185582 30588066 PMC6294061

[8] Carden MA Little J. Emerging disease-modifying therapies for sickle cell disease. Haematologica. (2019) 104:1710–9. 10.3324/haematol.2018.207357 31413089 PMC6717563

[9] Connes P Coates TD. Autonomic nervous system dysfunction: implication in sickle cell disease. C R Biol. (2013) 336:142–7. 10.1016/j.crvi.2012.09.003 23643396

[10] Coates TD Chalacheva P Zeltzer L Khoo MCK. Autonomic nervous system involvement in sickle cell disease. Clin Hemorheol Microcirc. (2018) 68:251–62. 10.3233/CH-189011 29614636

[11] Veluswamy S Shah P Denton CC Chalacheva P Khoo MCK Coates TD. Vaso-occlusion in sickle cell disease: is autonomic dysregulation of the microvasculature the trigger? J Clin Med. (2019) 8:1690. 10.3390/jcm8101690 31618931 PMC6832215

[12] Rogers VE Lewin DS Winnie GB Geiger-Brown J. Polysomnographic characteristics of a referred sample of children with sickle cell disease. J Clin Sleep Med. (2010) 6:374–81. 10.5664/jcsm.27880 20726287 PMC2919669

[13] Raghunathan VK Whitesell PL Lim SH. Sleep-disordered breathing in patients with sickle cell disease. Ann Hematol. (2018) 97:755–62. 10.1007/s00277-017-3199-z 29214337

[14] Willen SM Rodeghier M Rosen CL DeBaun MR. Sleep disordered breathing does not predict acute severe pain episodes in children with sickle cell anemia. Am J Hematol. (2017) 93:478–85. 10.1002/ajh.25013 29266432 PMC5842111

[15] Sharma S Efird JT Knupp C Kadali R Liles D Shiue K. Sleep disorders in adult sickle cell patients. J Clin Sleep Med. (2015) 11:219–23. 10.5664/jcsm.4530 25515282 PMC4346642

[16] Rogers VE Marcus CL Jawad AF Smith-Whitley K Ohene-Frempong K Bowdre C. Periodic limb movements and disrupted sleep in children with sickle cell disease. Sleep. (2011) 34:899–908. 10.5665/SLEEP.1124 21731140 PMC3119832

[17] Eckert DJ Younes MK. Arousal from sleep: implications for obstructive sleep apnea pathogenesis and treatment. J Appl Physiol. (2014) 116:302–13. 10.1152/japplphysiol.00649.2013 23990246

[18] Chalacheva P Ji Y Rosen CL DeBaun MR Khoo MCK Coates TD. Nocturnal peripheral vasoconstriction predicts the frequency of severe acute pain episodes in children with sickle cell disease. Am J Hematol. (2021) 96:60–8. 10.1002/ajh.26014 33027545 PMC8697370

[19] Berger RD Akselrod S Gordon D Cohen RJ. An efficient algorithm for spectral analysis of heart rate variability. IEEE Trans Biomed Eng. (1986) 33:900–4. 10.1109/TBME.1986.325789 3759126

[20] Cortes C Vapnik V. Support-vector networks. Mach Learn. (1995) 20:273–97. 10.1007/BF00994018

[21] Breiman L. Random forests. Mach Learn. (2001) 45:5–32. 10.1023/A:1010933404324

[22] Geurts P Ernst D Wehenkel L. Extremely randomized trees. Mach Learn. (2006) 63:3–42. 10.1007/s10994-006-6226-1

[23] Freund Y Schapire RE. A decision-theoretic generalization of on-line learning and an application to boosting. J Comput Syst Sci. (1997) 55:119–39. 10.1006/jcss.1997.1504

[24] LeCun Y Bengio Y Hinton G. Deep learning. Nature. (2015) 521:436–44. 10.1038/nature14539 26017442

[25] Le WT Maleki F Romero FP Forghani R Kadoury S. Overview of machine learning: part 2: deep learning for medical image analysis. Neuroimaging Clin N Am. (2020) 30:417–31. 10.1007/978-3-030-33128-3 33038993

[26] Friedman JH. Greedy function approximation: a gradient boosting machine. Ann Stat. (2001) 29:1189–232. 10.1214/aos/1013203451

[27] Friedman JH. Stochastic gradient boosting. Comput Stat Data Anal. (2002) 38:367–78. 10.1016/S0167-9473(01)00065-2

[28] Chen T Guestrin C. XGBoost: a scalable tree boosting system. In: Proceedings of the ACM SIGKDD International Conference on Knowledge Discovery and Data Mining, Vol. 13–17. Association for Computing Machinery (2016). p. 785–94. 10.1145/2939672.2939785

[29] Branco P Torgo L Ribeiro RP. A survey of predictive modeling on imbalanced domains. ACM Comput Surv. (2016) 49:31:1–31:50. 10.1145/2907070

[30] Davis J Goadrich M. The relationship between Precision-Recall and ROC curves. In: ICML '06: Proceedings of the 23rd International Conference on Machine learning. Pittsburgh, PA (2006). p. 233–40. 10.1145/1143844.1143874

[31] Saito T Rehmsmeier M. The precision-recall plot is more informative than the roc plot when evaluating binary classifiers on imbalanced datasets. PLoS ONE. (2015) 10:e0118432. 10.1371/journal.pone.0118432 25738806 PMC4349800

[32] Ravi D Wong C Deligianni F Berthelot M Andreu-Perez J Lo B. Deep learning for health informatics. IEEE J Biomed Health Inform. (2017) 21:4–21. 10.1109/JBHI.2016.2636665 28055930

[33] Lyon A Mincholé A Martínez JP Laguna P Rodriguez B. Computational techniques for ECG analysis and interpretation in light of their contribution to medical advances. J R Soc Interface. (2018) 15:20170821. 10.1098/rsif.2017.0821 29321268 PMC5805987

[34] Fiorillo L Puiatti A Papandrea M Ratti P Favaro P Roth C. Automated sleep scoring: a review of the latest approaches. Sleep Med Rev. (2019) 48:101204. 10.1016/j.smrv.2019.07.007 31491655

[35] Korkalainen H Aakko J Duce B Kainulainen S Leino A Nikkonen S. Deep learning enables sleep staging from photoplethysmogram for patients with suspected sleep apnea. Sleep. (2020) 43:zsaa098. 10.1093/sleep/zsaa098 32436942 PMC7658638

[36] Pombo N Garcia N Bousson K. Classification techniques on computerized systems to predict and/or to detect apnea: a systematic review. Comput Methods Programs Biomed. (2017) 140:265–74. 10.1016/j.cmpb.2017.01.001 28254083

[37] Mullin JE Cooper BP Kirkham FJ Rosen CL Strunk RC DeBaun MR. Stability of polysomnography for one year and longer in children with sickle cell disease. J Clin Sleep Med. (2012) 8:535–9. 10.5664/jcsm.2150 23066365 PMC3459199

[38] Espinoza J Shah P Veluswamy S Zeltzer L Khoo MCK Coates TD. Aura and mental stress are associated with reports of pain in sickle cell disease-a pilot study using a mobile application. Am J Hematol. (2020) 95:E101–3. 10.1002/ajh.25736 31957078 PMC7678711

[39] Fisher K Laikin AM Howard Sharp KM Criddle CA Palermo TM Karlson CW. Temporal relationship between daily pain and actigraphy sleep patterns in pediatric sickle cell disease. J Behav Med. (2018) 41:416–22. 10.1007/s10865-018-9918-7 29532199

