# Supplementary material for: Identifying Elevated Risk for Future Pain Crises in Sickle-Cell Disease Using Photoplethysmogram Patterns Measured During Sleep: A Machine Learning Approach
Source: Front Digit Health. 2021 Jul 26;3:714741. doi: 10.3389/fdgth.2021.714741 (PMC8360353; doi:10.3389/fdgth.2021.714741)
Supplement: Supplementary file 1 [file Data_Sheet_1.PDF]

SUPPLEMENTARY MATERIALS

Selection of Input Features: A more detailed account

We began with a total of 44 candidate features derived from 5 groups of information: (a) clinical data and subject characteristics, (b) sleep-related indices, (c) all-night RRI and heart rate variability indices, (d) all-night PPGa statistics, and (e) compact descriptors of the dynamic fluctuations in PPGa and RRI associated with detected vasoconstrictions. The screening procedure we applied was aimed at: (a)maximizing the association between each feature and pain category, and (b) minimizing the pairwise Spearman's correlation coefficient between that feature and other candidate features in the same feature group. The algorithm was as follows:

1. Compute the pairwise Spearman's correlation coefficient for all the features of the same feature group.
2. For each of the features, divide the subjects into the group with high-pain and the group with low-pain, and then perform the Wilcoxon Rank-Sum test on the two groups.
3. Put all features of the same type in the overall feature pool, and rank them according to their Wilcoxon Rank-Sum test p-values in ascending order.
4. While the overall pool is not empty, select the feature with lowest p-value. Move the selected feature from overall pool to the pool of selected features.
5. For all the features in the overall pool, remove the ones with absolute value of Spearman's rank correlation coefficient ≥ 0.5 with the feature selected in step 4.
6. Repeat Steps 4 and 5 until the overall pool is empty.
7. All the features in selected pool are used as inputs to the ML-based models. The candidate features and selected features are displayed in Table S1 below.

Table S1. List of all candidate features derived from data

Feature	Feature Group	Selected for Analysis?
Age	Clinical/subject	Yes
Sex	Clinical/subject	Yes
Hemoglobin	Clinical/subject	Yes
White blood cell	Clinical/subject	Yes
Reticulocyte	Clinical/subject	Yes
Neutrophil	Clinical/subject	Yes
Diastolic blood pressure	Clinical/subject	Yes
Systolic blood pressure	Clinical/subject	Yes
Body mass index	Clinical/subject	Yes
Hydroxyurea prescription	Clinical/subject	Yes
Arousal index	Sleep-related	Yes

AHI	Sleep-related	Yes
Number of limb movement	Sleep-related	Yes
Number of PLM sequences	Sleep-related	No
Effective sleep duration	Sleep-related	Yes
RRI SDNN full night	RRI & HRV-related	No
RRI SDANN full night	RRI & HRV-related	No
RRI SDNN index full night	RRI & HRV-related	No
RRI triangle index full night	RRI & HRV-related	No
Median of RRI mean per 5min	RRI & HRV-related	Yes
Median of RRI std per 5min	RRI & HRV-related	No
Median of RRI triangle index per 5min	RRI & HRV-related	No
Median of RRI RMSSD per 5min	RRI & HRV-related	No
Median of RRI LFP per 5min	RRI & HRV-related	No
Median of RRI normalized LFP per 5min	RRI & HRV-related	No
Median of RRI HFP per 5min	RRI & HRV-related	No
Median of RRI normalized HFP per 5min	RRI & HRV-related	No
Median of RRI LHR per 5min	RRI & HRV-related	No
Median of PPGa mean per 5min	PPGa-related	No
Median of PPGa std per 5min	PPGa-related	No
PPGa coefficient of variation (CV) full night	PPGa-related	No
Median of PPGa CV per 5min	PPGa-related	Yes
Median of PPGa LFP per 5min	PPGa-related	No
Median of PPGa HFP per 5min	PPGa-related	No
Avasoc: median of Avasoc for all vasoconstriction events	Vasoconstriction-related	No
Mvasoc: median of Mvasoc for all vasoconstriction events	Vasoconstriction-related	Yes
A_{RRI-}: median of A_{RRI-} for all vasoconstriction events	Vasoconstriction-related	Yes

A_{RRI+}: median of A_{RRI+} for all vasoconstriction events	Vasoconstriction-related	Yes
*A_{RRI}: median of A_{RRI} for all vasoconstriction events	Vasoconstriction-related	No
**R_{RRI+}: median of R_{RRI+} for all vasoconstriction events	Vasoconstriction-related	No
Tvasoc: median of Tvasoc for all vasoconstriction events	Vasoconstriction-related	No
Nvasoc: number of vasoconstriction events during sleep	Vasoconstriction-related	Yes
***Exposure index	Vasoconstriction-related	No
****Dosage index	Vasoconstriction-related	No

*A_{RRI} is defined as A_{RRI+} - A_{RRI-} of each vasoconstriction event.

**R_{RRI+} is ratio of A_{RRI+} over Tvasoc of each vasoconstriction event.

***Exposure index is defined as $\sum_{i=1}^{N_{\text{vasoc}}}\left(A_{\text{RRI}_i} - A_{\text{vasoc}_i}\right)$

****Dosage index is defined as $\frac{\sum_{i=1}^{N_{\text{vasoc}}}\left(A_{\text{RRI}_i} - A_{\text{vasoc}_i}\right)}{\sum_{i=1}^{N_{\text{vasoc}}} T_{\text{vasoc}_i}}$

Architecture of the CNN and MLP models used in this study

(1) CNN Model:

The structure of the CNN model we designed for this study is displayed below:

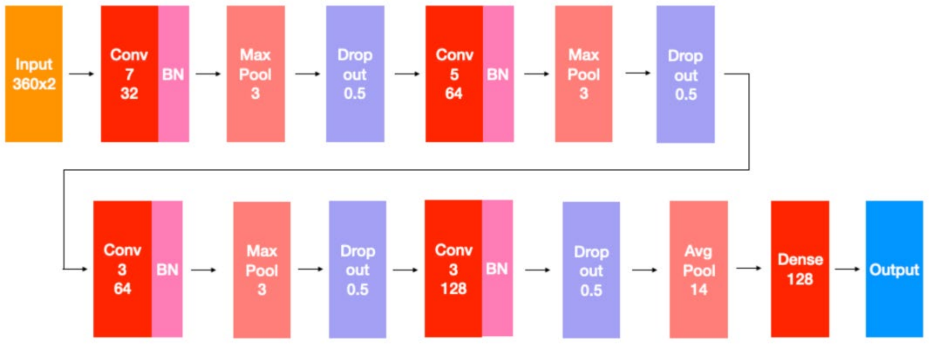

The input layer accepts 2 channels with 360 samples each, with one channel containing the PPGa time-series and the other channel containing the RRI time-series for each vasoconstriction segment (30 s of pre-vasoconstriction baseline plus 150 s during vasoconstriction, with sampling interval of 0.5 s).

Following the sequence of layers, "Conv, 7, 32" carries out 1D convolution operations with filter size 7 and 32 channels. Batch normalization ("BN") is used to accelerate the training process. "Max Pool 3" refers to a 1D max pool of size 3. "Dropout 0.5" represents the dropout layer which randomly discards 50% of the neurons during training process to prevent overfitting. This is followed by "Conv,5,64" with "BN", "Max Pool 3", "Dropout 0.5", then "Conv,3,64" with "BN", "Max Pool 3", "Droput 0.5", and "Conv,3,128" with "BN" and "Dropout 0.5", then "Avg Pool 14", the global average pool that outputs the average number of its input tensor (with length 14), and finally "Dense 128" which is a fully-connected layer with 128 neurons.

(2) MLP Model:

The MLP model is a fully-connected neural network with an input layer of size equal to number of input features, one hidden layer of size 100, and one output neuron of size 1, which outputs a probability score between 0 and 1.
